# BrainBrowser: distributed, web-based neurological data visualization

**DOI:** 10.3389/fninf.2014.00089

**Published:** 2015-01-13

**Authors:** Tarek Sherif, Nicolas Kassis, Marc-Étienne Rousseau, Reza Adalat, Alan C. Evans

**Affiliations:** McGill Centre for Integrative Neuroscience, McConnell Brain Imaging Centre, Montreal Neurological Institute, McGill UniversityMontreal, QC, Canada

**Keywords:** visualization, neuroimaging, neurology, WebGL, HTML5

## Abstract

Recent years have seen massive, distributed datasets become the norm in neuroimaging research, and the methodologies used to analyze them have, in response, become more collaborative and exploratory. Tools and infrastructure are continuously being developed and deployed to facilitate research in this context: grid computation platforms to process the data, distributed data stores to house and share them, high-speed networks to move them around and collaborative, often web-based, platforms to provide access to and sometimes manage the entire system. BrainBrowser is a lightweight, high-performance JavaScript visualization library built to provide easy-to-use, powerful, on-demand visualization of remote datasets in this new research environment. BrainBrowser leverages modern web technologies, such as WebGL, HTML5 and Web Workers, to visualize 3D surface and volumetric neuroimaging data in any modern web browser without requiring any browser plugins. It is thus trivial to integrate BrainBrowser into any web-based platform. BrainBrowser is simple enough to produce a basic web-based visualization in a few lines of code, while at the same time being robust enough to create full-featured visualization applications. BrainBrowser can dynamically load the data required for a given visualization, so no network bandwidth needs to be waisted on data that will not be used. BrainBrowser's integration into the standardized web platform also allows users to consider using 3D data visualization in novel ways, such as for data distribution, data sharing and dynamic online publications. BrainBrowser is already being used in two major online platforms, CBRAIN and LORIS, and has been used to make the 1TB MACACC dataset openly accessible.

## 1. Introduction

**Figure 1:**
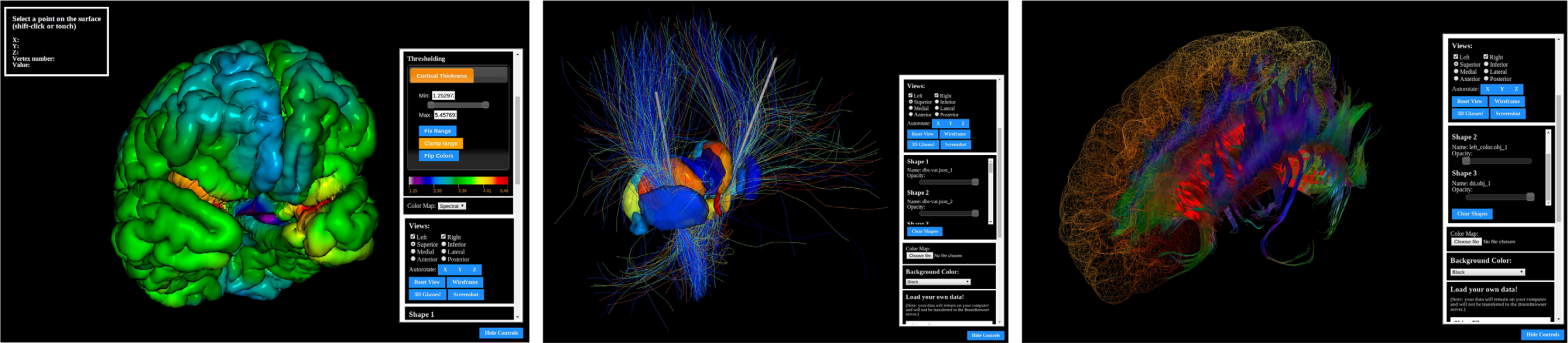
**The BrainBrowser Surface Viewer**.

**Figure 2:**
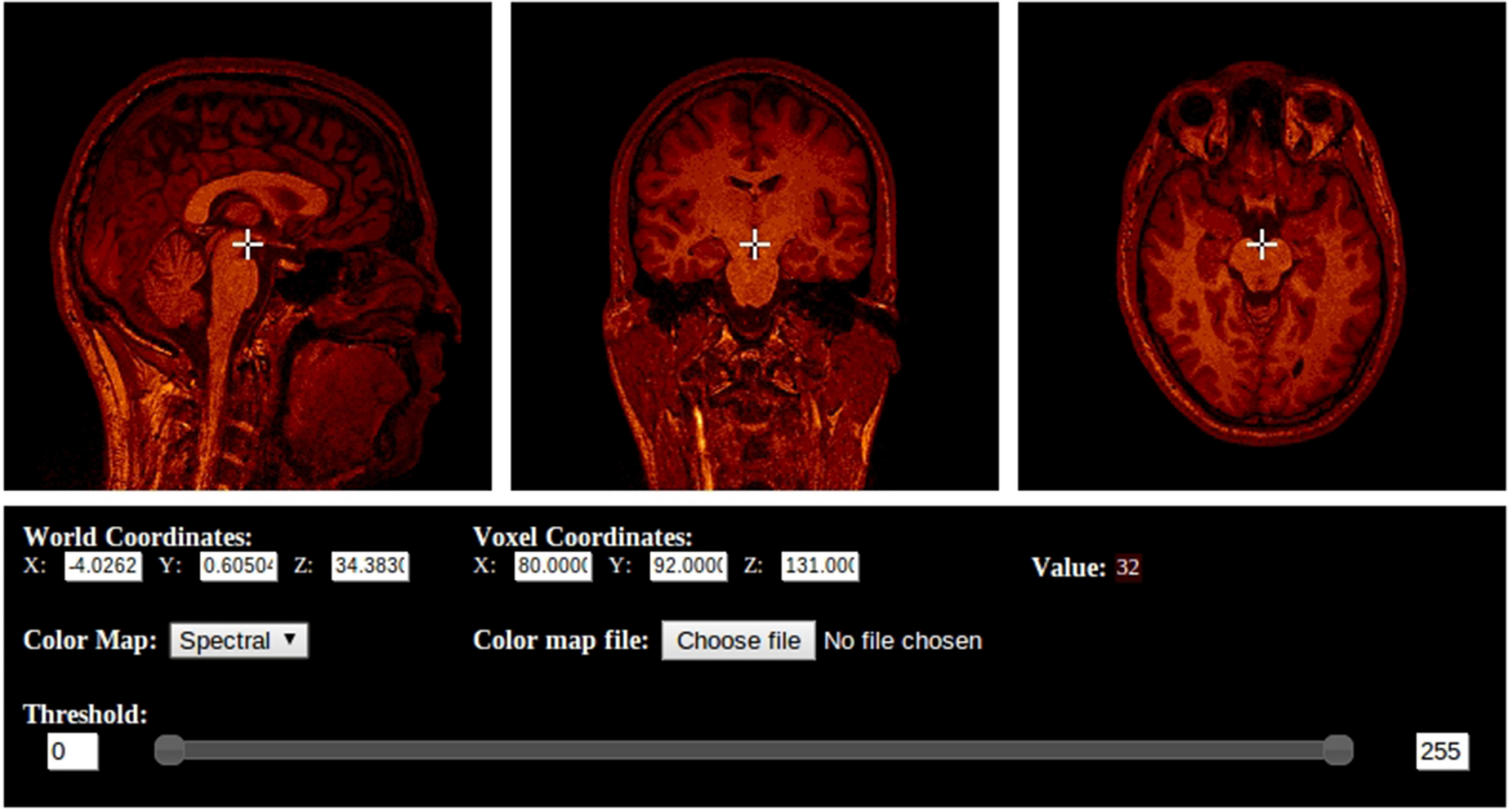
**The BrainBrowser Volume Viewer**.

BrainBrowser is an open source JavaScript library exposing a set of web-based 3D visualization tools primarily targeting neuroimaging. Using open web technologies, such as WebGL and HTML5, it allows for real-time manipulation and analysis of 3D imaging data through any modern web browser. BrainBrowser includes two major components. The BrainBrowser Surface Viewer (Figure 1) is a WebGL-based 3D viewer capable of displaying 3D surfaces in real time and mapping various sorts of data to them. The BrainBrowser Volume Viewer (Figure 2) is an HTML5 Canvas-based viewer allowing slice-by-slice traversal of 3D or 4D MINC volumetric data (Vincent et al., 2004).

In recent years, neuroimaging research has seen itself inundated by large, distributed datasets that have necessitated a shift in how scientists approach their research: guiding hypotheses are often articulated after analyzing the mass of available data (Margulies et al., 2013), and data sharing has become a necessity for scientific discovery (Jomier et al., 2011). Several large-scale, distributed, collaborative platforms have been developed to facilitate this new approach, and they tend to integrate poorly with traditional visualization tools requiring a local installation and local data. These tools and their dependencies would have to be installed locally, and data would generally have to be exported from the platform in order to be visualized in the local environment. Web-based visualization tools, on the other hand, present significant benefits in the context of distributed research platforms:

- They can be easily integrated into existing web-based platforms.
- Other than the web browser, no software or libraries need to be installed.
- If a visualization doesn't require the entirety of a remote dataset at once, it can load required data on-demand, potentially saving bandwidth.

Ideally, a web-based visualization tool might have the following properties:

- **Performant and responsive:** If network latency or lagging performance interfere with usability, researchers will not use it.
- **Doesn't require any browser plugins (e.g., Java, Flash):** Plugins add extra friction to the deployment of tools that depend on them. They require users to install software on their machines and keep it up to date. Furthermore, not all users have the administrative access to their machines that is required to install most plugins.
- **Dynamic and interactive:** If the tool is to be used to explore data, it should allow that data to be loaded, removed, manipulated and modified with minimal effort.
- **Extensible:** Many labs use different data formats, standards and requirements can change rapidly, so the tool should be able to adapt.
- **Open source:** It's easier for researchers to trust the results they're seeing if they can verify how the tool is functioning. If need be, they can also extend, customize and tweak the tool to fit their needs.

While this shift in research requirements has been taking place, modern web standards have introduced a host of new technologies that are built directly into modern web browsers and allow for the creation of high-performance, web-based applications that rival much of what is available offline. Improvements to the JavaScript language itself and the optimizations made by browser vendors such as Google and Mozilla to their JavaScript engines have created a base on which robust applications can be built. At the same time, Graphical Processing Unit (GPU) access through WebGL, and multi-threaded processing through Web Workers, blur the lines between what is possible on native versus web applications.

The fact that these technologies are now a part of the hyper-connected web platform has also made it possible to consider using visualization technologies in novel ways:

- **Publication:** With scientific articles now being published online, publishers are looking for ways to disseminate datasets and present them in more meaningful ways (Jomier et al., 2011). It is now possible to replace the static visual media of traditional print publications, such as static figures or charts, with more dynamic, interactive 2D or 3D visualizations^1^.
- **Distribution:** Online visualization tools can also be used as a means to distribute data, as researchers can use the tools to explore a remote dataset directly. BrainBrowser, for example, was used to share the MACACC dataset, as will be discussed further in Section 4.1. TissueStack, which will be discussed in Section 2, was used for remote visualization of the BigBrain dataset (Amunts et al., 2013) upon its release.

As will be discussed in Section 2, approaches to using the aforementioned web technologies for visualization can be broadly split into two categories. The first requires some back-end infrastructure to support a front-end client. This infrastructure will usually involve some processing of the data to be served, and might also include some proxying or database support. The second runs completely in the browser, requiring the back end to do nothing more than serve static files. BrainBrowser takes the latter approach for the following reasons:

1. **Scalability:** Both approaches require that the resources available on the server be capable of handling the user load at all times, but a fully front-end application puts a much lighter load on those resources. For back-end infrastructure, growth of the user base necessitates an expansion of infrastructure resources to prevent the application from blocking. In a commercial setting, where new users bring increased revenues, this type of expansion might not be considered problematic. In a research setting, however, where the relationship between users and revenue is not so direct, the cost of scaling becomes a major concern.
2. **Network latency:** If simple manipulations of a visualization require continuous network traffic, the responsiveness of the application will be bound by network latency. After the initial load of the data to be visualized, most fully front-end applications will no longer be dependent on the network.
3. **Flexibility:** Being independent of any particular server allows a fully front-end application to pull its data from essentially anywhere. An application could allow users visualize data they have stored locally on their machines without being required to upload them to a server. There is also nothing preventing fully front-end application from requesting data from a server with more elaborate infrastructure, thus allowing it to benefit from both approaches.

BrainBrowser has been developed to facilitate the exploitation of modern web technologies for a wide range of purposes. It exposes an application programming interface (API) that is simple enough to create a basic, interactive visualization in a few lines of code, while being deep enough to build more complex visualizations involving dynamic, on-demand fetching and loading of remote data. It uses WebGL, the HTML5 Canvas and Web Workers to push as much of the processing client-side as possible, thus minimizing the effect of network lag on its usage and eliminating the need for browser plugins. BrainBrowser is an open-source project, meaning that its users can verify the code directly and even modify it to fit their needs.

## 2. Related work

Early attempts at advanced web-based visualization required plugins of one form or another, as standard web technologies could not provide the functionality and performance required for more advanced applications. The Java Internet Viewer (JIV, Cocosco and Evans, 2001) is an example of this type of solution. JIV could run as a standalone application or as a Java applet embedded in a web page. JIV presented the user with three orthogonal slices of a MINC volume that could be navigated by manipulating a cursor on each of the slices.

As more advanced web standards were proposed and implemented, many research groups began to show an interest in exploiting the new robustness of the web platform for various forms of advanced visualization. Google Body (now Zygote body, Blume et al., 2011) was an early example of what had become possible. Google Body renders several layers of a human body, from muscle tissues to blood vessels, and allows layers to be manipulated (e.g., by toggling their visibility or rendering them transparently) to facilitate viewing.

In the domain of neuroimaging, several tools have been developed, each offering a different approach to the problem of visualization. We present here a few examples that we believe exemplify the two approaches, back-end infrastructure and fully front-end, that were discussed in Section 1:

**ParaViewWeb** (Jourdain et al., 2011) functions as a web client for ParaView^2^, an open-source, server-side, parallel data visualization framework. ParaView uses the Visualization Toolkit^3^ (VTK) to generate visualizations on the server that the ParaViewWeb client interacts with through a web service proxy.
**TissueStack**^4^ (Lin et al., 2013) is an open-source application for visualizing MINC and NIfTI (Cox et al., 2004) files on the web. It is composed of an image server that tiles the volume to be visualized, a configuration database and an HTML5-based client for rendering the tiles.
**The X Toolkit**^5^ (XTK, Hähn et al., 2014) is an open-source, front-end JavaScript library that, unlike the previous two applications, does not require any back-end infrastructure. Images can be loaded directly into the browser and rendered using WebGL. XTK supports volume rendering, as well as cross-sectional slicing that can be navigated programmatically. It allows labels and colors to be mapped to a surface and can also perform lower-level geometric functions such as constructive solid geometry.
**Papaya**^6^, like XTK, is an open-source visualization tool that operates completely in the browser. Unlike XTK, however, Papaya is implemented as an independent application rather than a library. It uses the 2D HTML5 Canvas API to implement an orthogonal viewer for NIfTI volumetric data that can be navigated using the keyboard or mouse. Users can manipulate the color mapping and thresholding of the intensity data being displayed.

## 3. Materials and methods

### 3.1. Core technologies

The **HTML5 Canvas element** is a high-performance, scriptable 2D drawing surface. Originally introduced by Apple in their Safari browser, it was eventually standardized by the Web Hypertext Application Working Group^7^ (WHATWG). The canvas element provides both a 2D drawing context exposing an API for drawing basic 2D shapes and images, and a WebGL context exposing an API for high-performance 3D graphics. The key aspect of the 2D API used by BrainBrowser is a set of functions for pixel-level image processing and manipulations. The Volume Viewer makes heavy use of this functionality to colorize and render volume slices to the screen.

**WebGL** is a low-level JavaScript graphics API that makes use of the HTML5 Canvas element to provide web pages with access to the GPU of the client computer. WebGL provides a platform for high-end, web-based 3D graphics programming without requiring any browser plugins. The API consists of control code written in JavaScript and GLSL shader code which runs directly on the GPU. WebGL is designed and maintained by the Khronos Group^8^. The BrainBrowser Surface Viewer makes use of WebGL through the three.js JavaScript library.

**Three.js**^9^ is a lightweight, open-source JavaScript library developed to abstract away much of the complexity of using the WebGL API directly. It implements a scene graph and allows one to manipulate the scene using intuitive constructs such as objects, lights and cameras, rather than the more mathematical buffers and matrices of the raw WebGL API. Three.js also provides intuitive APIs for more complex operations such as ray casting. The BrainBrowser Surface Viewer was built using three.js.

**Web Workers** are a recent standard adopted by the World Wide Web Consortium^10^ (W3C) and WHATWG providing an API to run multiple JavaScript processing threads concurrently in the same browser. Prior to the introduction of Web Workers, one of the glaring weaknesses of JavaScript, when it came to building performance-intensive applications, was that it was designed to be a single-threaded language. As such, all processing, rendering and event-handling code had to share the same processing thread, even if running on a multi-core computer. BrainBrowser makes extensive use of Web Workers to parse large data files in threads that are separate from the main UI and rendering threads, allowing the interface to remain responsive to the user while these heavy operations are being performed.

### 3.2. BrainBrowser

At its core, BrainBrowser is a JavaScript library that can be inserted into an ordinary web page. It exposes an intuitive API to the developer that is simple enough to begin visualizing a dataset in 5–10 lines of JavaScript code, while at the same time being robust enough perform more complex color mapping, thresholding or blending of datasets.

Much of this flexibility comes from the modular approach taken in BrainBrowser's design. The library has been built from several independent, interconnected layers. The BrainBrowser core contains modules and functions related to functionality required by both the Surface Viewer and Volume Viewer: data loading over the network or from the file system, color mapping of intensity data, event handling, data storage and various utility functions. Aside from this shared core, the Surface Viewer and Volume Viewer each contain modules encapsulating more specific functionality.

#### 3.2.1. Surface viewer

**Figure 3:**
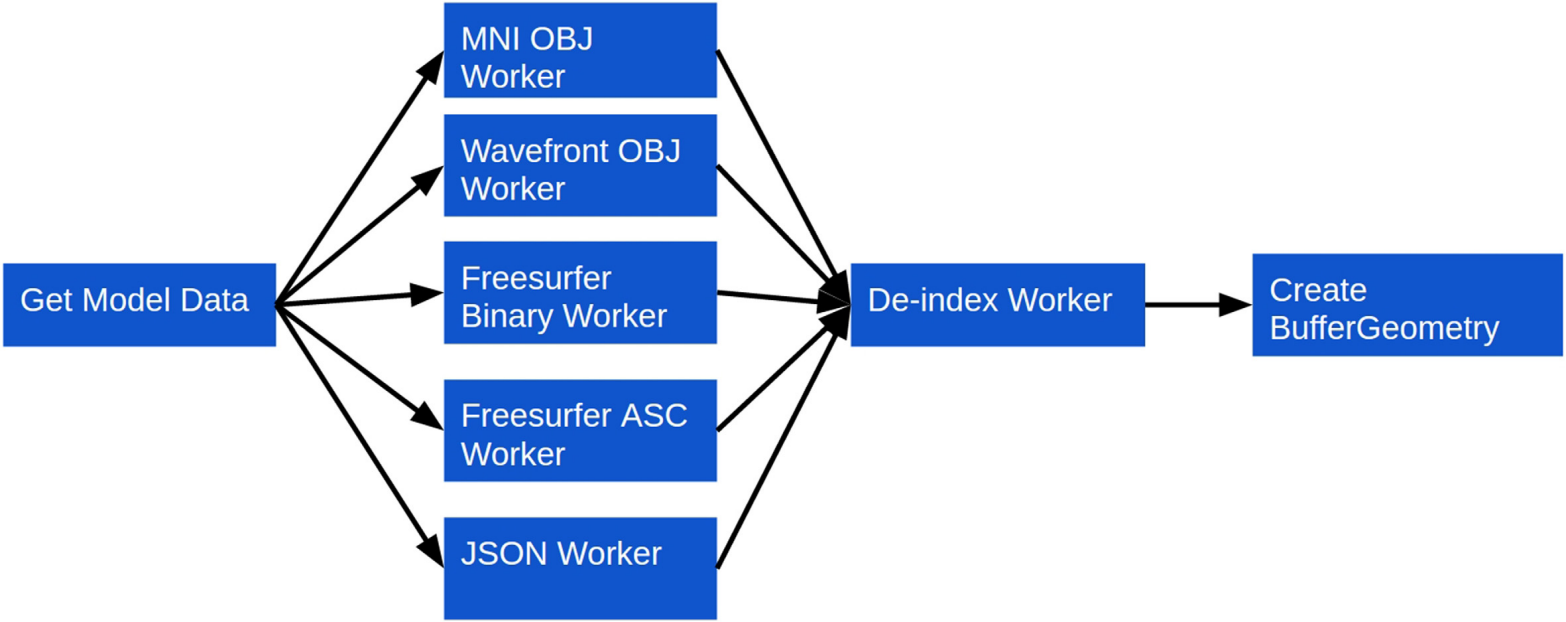
**Surface Viewer workflow for loading surface geometry**.

Rendering a model using the BrainBrowser Surface Viewer requires only a few method calls (Listing 1), but behind these calls are several layers interacting to optimize performance (Figure 3):

1. Model geometry data are loaded asynchronously over the network using AJAX^11^, or from the file system using the FileReader JavaScript API^12^. Geometry data can be described in several parts, depending on the data format, and these will be parsed as different shapes in the Surface Viewer object model. Different shapes can be manipulated either individually or collectively (see item 4). Data can be in one of several supported binary or text formats commonly used in neuroimaging research: MNI OBJ^13^, Freesurfer binary and Freesurfer ASC^14^. The Surface Viewer also supports Wavefront OBJ^15^, as well as a custom JSON^16^-based format we developed to facilitate exporting surfaces out of external applications and into the Surface Viewer. Supporting these formats directly, rather than requiring they be converted beforehand, is what allows the Surface Viewer to remain independent of any server-side infrastructure that would be necessary to preprocess them.
2. Geometry data are sent to one of several Web Workers for parsing. Each supported file type in the Surface Viewer is associated with a Web Worker that can be spawned to convert a given geometry description into the geometry object model that the Surface Viewer uses internally (Listing 2). This architecture, in which one Web Worker is responsible for each data format, exposes a plugin framework that can be used to add support for other data formats. Adding support for a new format requires only the creation of a Web Worker script that can convert the new format into the Surface Viewer object model.
3. Once data are converted to the Surface Viewer object model, a final step is required before the data can be displayed. One of the weaknesses of the current WebGL specification is that indices describing how to build a mesh from individual vertices are limited to 16 bits in size. This puts a limit of 65536 vertices on indexed models in WebGL. The Surface Viewer is meant to handle datasets that fall well outside this limit (the DTI sample on the BrainBrowser website^17^, for example, contains 560674 vertices). To get around this problem, BrainBrowser will send model data to a second Web Worker that de-indexes the model data, essentially “unrolling” the indices so that simply traversing the list of vertices is sufficient to draw the model.
4. Once model data are prepared, they are passed to a high-performance three.js BufferGeometry object to prepare them for display. The Surface Viewer supports displaying models made out of polygons (used for most surfaces) or lines (used for tractography data). If normals or vertex colors are provided in the model description, they will also be passed to the BufferGeometry. Otherwise, the vertex colors will be set to gray, and the normals will be inferred from the geometry using available three.js utility methods. If several shapes were described in the input data, each shape will get its own BufferGeometry object. This allows each shape to be manipulated individually (to apply different opacity levels to different parts, for example) or collectively (for rotating the model as a whole, for example).

**Listing 1:**
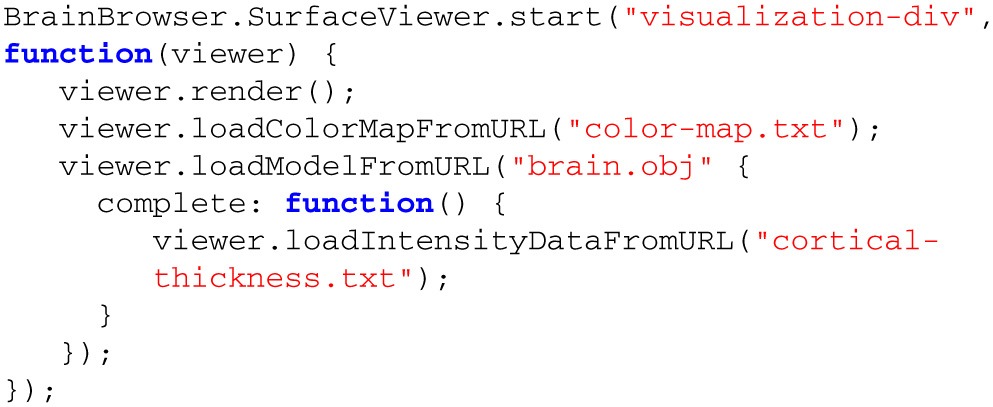
**BrainBrowser API code to create an instance of the Surface Viewer and use it to load and display surface and intensity data**.

**Listing 2:**
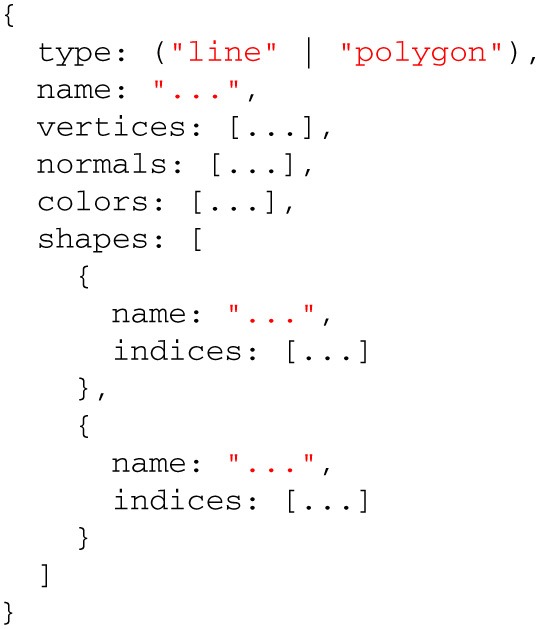
**The BrainBrowser Surface Viewer object model**.

**Figure 4:**
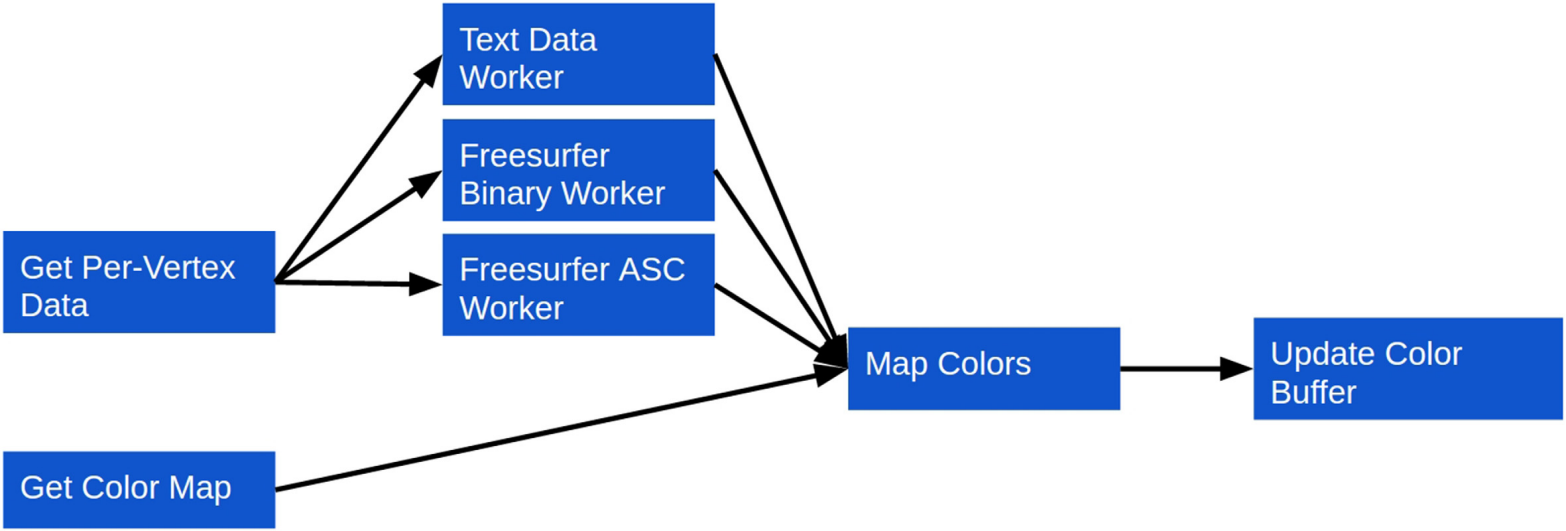
**Surface Viewer workflow for coloring a surface based on per-vertex intensity data**.

If per-vertex intensity data are to be mapped to a loaded model, three additional steps are taken (Figure 4):

1. A color map file is loaded. Similarly to the model data, this can be either over the network or from the local file system. Since parsing color map files is an inexpensive operation, however, it is done directly in the main thread, rather than in a Web Worker.
2. The intensity data are loaded and parsed. Loading, as with the other forms of data described, can occur over the network or from the local file system. Parsing is done similarly to the geometry data, with separate Web Workers being implemented for each supported intensity data type. As with the geometry, this creates a plugin framework for intensity data support, making it straightforward to add support for new formats. Currently, plain-text, Freesurfer binary and Freesurfer ASC intensity data formats are supported.
3. The color map is used to assign a color to the intensity scalar associated with each vertex. The mapped colors are then applied to the BufferGeometry color buffer, which updates the model's colors on a per-vertex basis.

Once a model is loaded and colorized, it can be manipulated in several ways:

- Intuitive mouse and touch controls to rotate, pan and zoom.
- A wireframe view that provides a clearer view of a model's geometry.
- Setting opacity on different parts of the model to reveal internal elements.
- Loaded intensity data can be manipulated in various ways such as setting intensity thresholds for color mapping, blending multiple datasets and programmatically modifying the dataset itself.

A key feature of the Surface Viewer is the picking mechanism that it implements. A simple method call that takes arbitrary x and y coordinates on the canvas (defaulting to the current mouse position) will return information about the vertex rendered closest to the given point. Returned data includes:

- The index of the vertex in the list of vertices.
- The x, y, and z coordinates of the selected vertex.
- The specific shape in the model that contains the selected vertex.

This picking mechanism makes it possible to implement more complex interactions with a rendered model based on the specific vertex with which a user is interacting. The MACACC dataset viewer that will be discussed in Section 4.1, for example, uses picking to dynamically load intensity data based on the selected vertex.

The Surface Viewer also implements an annotation system that is built on top of the picking mechanism. Annotations allow a user to select a specific vertex and associate arbitrary data with it: links, images, charts, text, etc. The annotation is rendered as a colored dot on the model, and the user can later click on the dot to retrieve the annotation. Internally, annotations are stored in a basic JavaScript object that can easily be converted to JSON for persistent storage in a back-end database.

#### 3.2.2. Volume viewer

**Figure 5:**
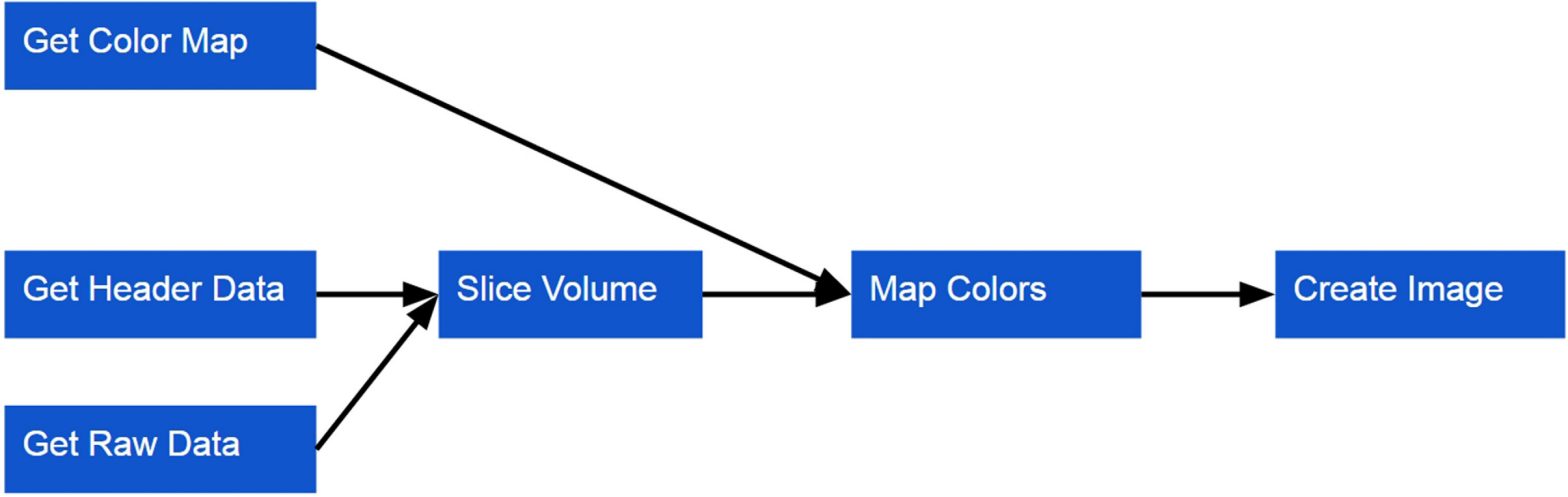
**Volume Viewer workflow for loading a volume**.

Like the Surface Viewer, the Volume Viewer only requires a few simple method calls to render a volume to the screen (Listing 3). Behind the scenes, the following steps are taken (Figure 5):

1. A color map is loaded via the network or the local file system. Unlike the Surface Viewer, the Volume Viewer requires the color map to be loaded beforehand as the volume is pure scalar intensity data, without any explicit geometry, so there can be no visualization without a color map.
2. A volume is loaded over the network or from the local file system. This data will be loaded in two parts: (a) the raw intensity data, (b) header information describing the dimensions of the data and relationship between the voxel and world coordinate spaces. Currently, only MINC data are directly supported, but similarly to the Surface Viewer, volume preparation is done with a plugin architecture that makes it straightforward to add support for other formats.
3. The color map is used to assign a color to the intensity scalar associated with each voxel. These colors are used the create the image rendered for a given slice through the volume.
4. Other options can also be set, including setting the panel size and creating an overlay view of the loaded models.

**Listing 3:**
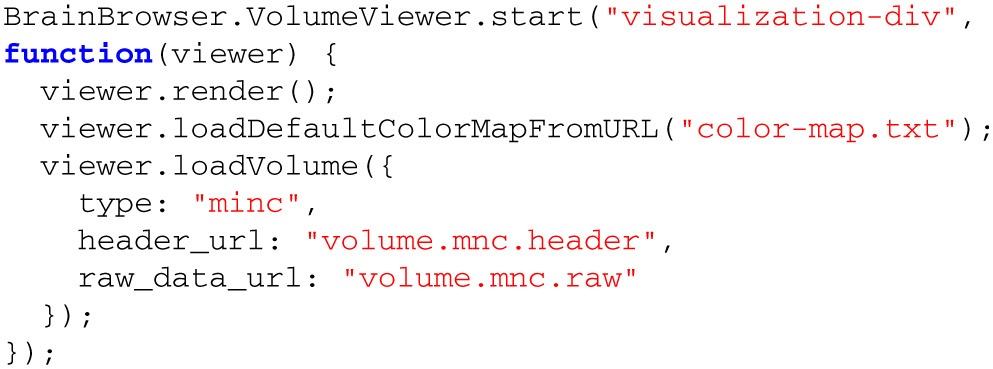
**BrainBrowser API code to create an instance of the Volume Viewer and use it to load and display a MINC volume**.

Once a volume is loaded, three orthogonal slices of the volume along the sagittal, transverse and coronal planes will be displayed on three separate canvases. Each canvas also renders a cursor indicating the current display position of the volume along the given plane. The view of the volume can be manipulated in the following ways:

- Moving the cursor using mouse or touch controls updates the displayed orthogonal slices in real time.
- If the volume is four-dimensional, the time dimension can be manipulated programmatically.
- Keyboard controls are also available for all dimensions to facilitate more precise navigation through the volume.
- The current position of cursor in the in volume can be retrieved in both voxel and world coordinates. The Volume Viewer provides methods that perform the conversion automatically.
- The distance in world coordinates between two points can be measured.
- Intensity thresholds can be set to focus on particular ranges.

If more than one volume is loaded, then the following functionalities also become available:

- The volumes can be combined into an overlaid view for comparison. The blending weights for each volume can be set to favor one or the other.
- The cursors can be synchronized across volumes to facilitate comparisons.

Any of these functionalities can be hooked into standard user interface callbacks for events such as clicks or text input to create more complex applications. To simplify the creation of such user interfaces when multiple volumes are loaded, the Volume Viewer also provides a templating mechanism that allows a single UI template to be defined and repeatedly instantiated for multiple volumes. The developer simply creates a template using standard HTML. When loading the volumes, the Volume Viewer fetches the template, instantiates the UI and then inserts the display canvases at the appropriate location.

## 4. Results

BrainBrowser has already been deployed for web-based visualization on a variety of platforms. Here, we will discuss three cases that we believe highlight BrainBrowser's strengths.

### 4.1. The MACACC Dataset

**Figure 6:**
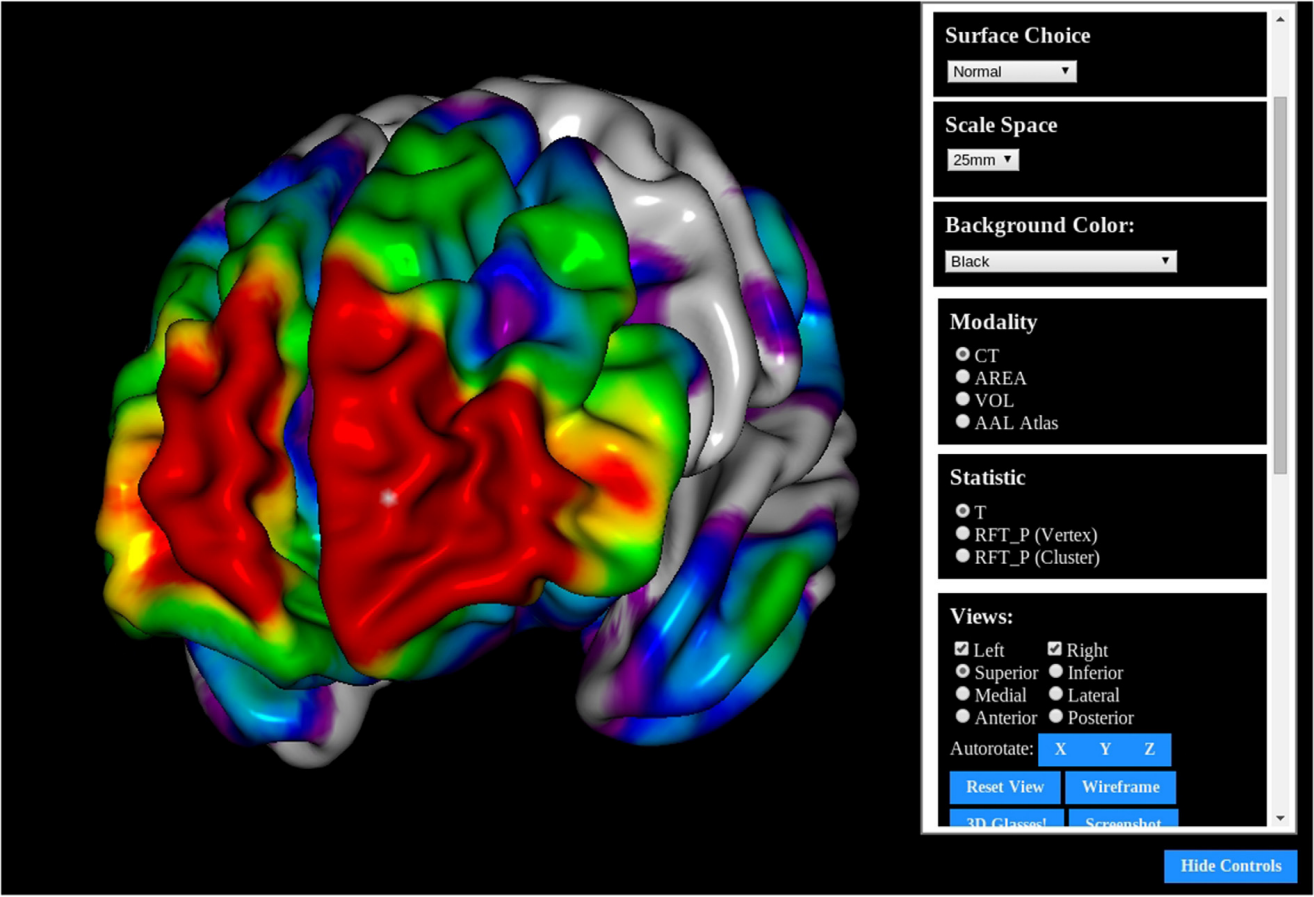
**The MACACC Viewer: A viewer built using the BrainBrowser Surface Viewer to visualize the MACACC dataset (Lerch et al., 2006)**.

The MACACC Dataset (Lerch et al., 2006) is a database of structural correlations across the cortex derived from the International Consortium for Brain Mapping (Mazziotta et al., 2001). Cortical thickness at each of 81924 3D locations were calculated using the CLASP algorithm (Kim et al., 2005). The MACACC Dataset contains maps for all cortical vertices for each of three vertex-wise morphological variables: thickness, area and volume. The total number of permutations encoded in the dataset is 81924 vertices × 9 blurring kernels × 3 morphological indices × 3 statistical indices for a total of 6.3 million data maps, requiring over 1TB of storage space. The MACACC dataset presents an ideal use case for BrainBrowser in that its size makes it extremely inconvenient to distribute or to visualize using traditional, locally installed tools. These tools would generally necessitate the transfer of the entire dataset to the machine on which it is to be visualized. BrainBrowser, on the other hand, makes it trivial to explore this dataset in an intuitive manner. The MACACC Viewer^18^ (Figure 6) uses the picking mechanism described in Section 3.2.1 to fetch vertex information based on the point where the user clicks on the screen. This vertex information is used to make a network request for the correlation map associated with the selected vertex and then use it to colorize the model. This dynamic fetching means that only the portion of the 1TB MACACC dataset associated with the selected vertex (approximately 455kB) is transferred over the network, thus minimizing bandwidth usage and increasing accessibility.

### 4.2. CBRAIN

CBRAIN (Sherif et al., 2014) is a distributed, collaborative, web-based, grid computing platform developed at McGill University. CBRAIN has been in active production since 2009 and currently has over 250 users from 53 cities in 21 countries around the world. CBRAIN is a complex system comprised of many interconnected components, but there are four components in particular that are relevant to the discussion here:

- Users interact with CBRAIN through a web interface that can be accessed through any modern web browser.
- Data are connected to CBRAIN through Data Providers, which are essentially storage devices that might be at any controlled, secure, network-accessible location.
- CBRAIN is connected to several High Performance Computing Centers (HPCs) at several locations around the world.
- Installed on the HPCs are data-processing tools that researchers use to analyze or process their data.

The current production deployment of CBRAIN consists of 11 HPCs in Canada, Germany and Korea. CBRAIN provides over 100TB of storage on its central servers, and several external Data Providers for specific projects or institutions are also registered with the system. A typical workflow for a CBRAIN user might include the following steps:

1. Register data with CBRAIN. This can be done by either creating a new Data Provider or by uploading data to one that already exists.
2. Visualize data as a preparatory quality control step before submitting them to an HPC.
3. Submit a job to an HPC through the web interface. This typically involves selecting the processing tool one wishes to use and the HPC on which to run the job.
4. Once the job is complete, results are saved back to the user's account.
5. Visualize results as a means of ensuring their quality or otherwise interpreting them.

Early in CBRAIN's development, visualizing data for the second and final steps was found to be problematic. It would often require downloading the data to visualize them locally. The Java Image Viewer (JIV) was used for a time to allow users to view images online. Among other problems, the requirement that users install a Java plugin on their machine was an issue for the reasons outlined in Section 1. BrainBrowser was an ideal solution in this situation as it could be integrated directly into the web-based UI of the platform without requiring a plugin. The Volume Viewer has now completely replaced JIV for visualizing MINC files in CBRAIN, and the Surface Viewer is now used to visualize surface files produced by the CIVET processing pipeline (Ad-Dab'bagh et al., 2006).

### 4.3. LORIS

LORIS (Das et al., 2011) is a web-based database system providing infrastructure to automate the flow of clinical data for complex multi-site neuroimaging studies. Initially developed to manage data for the NIH MRI Study of Normal Brain Development (Evans, 2006), LORIS has since been adapted and implemented in numerous decentralized large-scale studies internationally, such as 1000BRAINS^19^ (Caspers et al., 2014, Germany), IBIS^20^ (USA), NeuroDevNet^21^ (Canada), GUSTO^22^ (Singapore) and NIHPD^23^ (USA).

As an example of the type of data LORIS handles, the NIH MRI Study of Normal Brain Development (NIHPD) used LORIS to house and distribute 3TB of native and processed data for over 2000 MRI acquisitions. Medical doctors visualized this data throughout the project to assess the quality of incoming scans. As with CBRAIN, visualization using traditional tools was problematic for users of LORIS, and like CBRAIN, JIV was used for a time as a solution that was imperfect for the reasons mentioned above. Currently, MINC volume files are visualized in LORIS using the Volume Viewer, and the Surface Viewer is used to visualize any compatible data that are stored in the system.

## 5. Discussion

The advent of big data in neurological research, and the new methodological approaches it necessitates, have lead to the development of large-scale, distributed platforms to facilitate its exploration. Modern web technologies such as HTML5 and WebGL, have made it possible to create web-based tools that integrate seamlessly into these new environments. Conversely, newly available technologies have opened doors to novel ways of using research data that were not previously possible. Interactive figures in online articles and online data exploration are two examples of such novel usage that were presented here.

BrainBrowser presents itself as a lightweight, flexible means to easily exploit modern web technologies for a variety of uses. A simple visualizer with full interactivity and touch controls can be embedded in a web page with just 5–10 lines of JavaScript code. On the other hand, the robustness of BrainBrowser can be exploited to create a full visualization user interface with controls to load intensity data, switch color mappings, set intensity thresholds, etc. As shown in the MACACC Viewer example, even more complex interactions can be built on top of BrainBrowser, with data being loaded interactively to allow for dynamic exploration of very large datasets.

Compared to the other web visualization solutions presented in Section 2, BrainBrowser differentiates itself on several fronts. BrainBrowser operates completely in the browser, which sets it apart from back-end infrastructure solutions like ParaViewWeb and TissueStack. A comparison of BrainBrowser to XTK and Papaya, the two other fully front-end solutions that were discussed, is given in Table 1.

**Table 1:** **Feature comparison between BrainBrowser, XTK and Papaya**.

	**View surfaces**	**View volumes**	**Web workers**	**Picking**	**Annotations**	**Plugin data format support**	**Load local files**
BrainBrowser	✓	✓	✓	✓	✓	✓	✓
XTK	✓	✓				✓	
Papaya		✓					✓

BrainBrowser's modular construction, and the fact that its source code is freely available, make it easily extensible and modifiable to suit the needs of its users. New data-parsing Web Workers can easily be added to BrainBrowser to allow it to support new file formats. This, in fact, ties into one of the major goals for the future of BrainBrowser: to continue building our open-source development community^24^ so it can directly develop the features it requires, including support for new file formats.

Moving forward, we would like to explore using BrainBrowser to visualize larger datasets (the BigBrain dataset, for example) that are not as granular as the MACACC dataset but are too large to be loaded directly into the browser. This would likely entail connecting a BrainBrowser-based application to a server that performs more back-end processing of the data, as was discussed in Section 1. There is also an enormous space for collaborative data exploration that has yet to be investigated. Using new technologies such as Web Sockets^25^ or WebRTC^26^, it is possible to create real-time collaborative environments that will facilitate the exploration of shared data in ways that have been impossible until now.

### 5.1. Data sharing

All source code for BrainBrowser is freely available on GitHub^27^ under the GNU Affero General Public License v3^28^. Demonstrations of BrainBrowser functionality are available on the BrainBrowser website^29^.

### Conflict of interest statement

The authors declare that the research was conducted in the absence of any commercial or financial relationships that could be construed as a potential conflict of interest.

## Footnotes

^1^For example: http://onpub.cbs.mpg.de/role-brocas-area-language.html

^2^http://www.paraview.org/

^3^http://vtk.org/

^4^https://github.com/NIF-au/TissueStack

^5^http://goxtk.com

^6^https://github.com/rii-mango/Papaya/

^7^https://whatwg.org/

^8^http://www.khronos.org/

^9^http://threejs.org/

^10^http://www.w3.org/

^11^https://developer.mozilla.org/en-US/docs/AJAX

^12^https://developer.mozilla.org/en-US/docs/Web/API/FileReader

^13^http://www.bic.mni.mcgill.ca/users/mishkin/mni_obj_format.pdf

^14^http://www.grahamwideman.com/gw/brain/fs/surfacefileformats.htm

^15^http://en.wikipedia.org/wiki/Wavefront_.obj_file

^16^https://developer.mozilla.org/en-US/docs/Glossary/JSON

^17^https://brainbrowser.cbrain.mcgill.ca/surface-viewer#dti

^18^https://brainbrowser.cbrain.mcgill.ca/macacc-viewer

^19^http://www.fz-juelich.de/inm/inm-1/EN/Forschung/1000_Gehirne_Studie/1000_Gehirne_Studie_node.html

^20^http://www.ibis-network.org

^21^http://www.neurodevnet.ca

^22^http://www.gusto.sg

^23^http://pediatricmri.nih.gov/nihpd/info/index.html

^24^https://github.com/aces/brainbrowser/graphs/contributors

^25^https://developer.mozilla.org/en/docs/WebSockets

^26^https://developer.mozilla.org/en-US/docs/Web/Guide/API/WebRTC

^27^https://github.com/aces/brainbrowser

^28^http://www.gnu.org/licenses/agpl-3.0.html

^29^https://brainbrowser.cbrain.mcgill.ca
